# What Predicts Patients’ Adoption Intention Toward mHealth Services in China: Empirical Study

**DOI:** 10.2196/mhealth.9316

**Published:** 2018-08-29

**Authors:** Zhaohua Deng, Ziying Hong, Cong Ren, Wei Zhang, Fei Xiang

**Affiliations:** ^1^ School of Medicine and Health Management Huazhong University of Science and Technology Wuhan China; ^2^ Department of Medical Records Room Affiliated Central Hospital of Zhengzhou University Zhengzhou China

**Keywords:** mHealth service, perceived risk, trust, TAM, Chinese patients, mobile phone

## Abstract

**Background:**

With the increasing concerns about the health of individuals in China and the development of information technology, mHealth enables patients to access health information and interact with doctors anytime and anywhere. Examining patients’ willingness to use mHealth is considered critical because its success depends on the adoption of patients.

**Objective:**

The objective of our study was to explore the determinants of mHealth service adoption among Chinese patients using an extended technology acceptance model (TAM) with trust and perceived risks.

**Methods:**

We conducted a questionnaire-based survey in 3 large hospitals in China and analyzed the data using structural equation modeling.

**Results:**

The results corroborated that the proposed model fits well. Trust, perceived usefulness, and perceived ease of use positively correlated with mHealth service adoption. Privacy and performance risks negatively correlated with the patients’ trust and adoption intention toward mHealth services. In addition, patients’ age and chronic diseases can help predict their trust level and adoption intention toward mHealth, respectively.

**Conclusions:**

We concluded that the TAM generally works in the context of mHealth adoption, although its significance has declined. In addition to technical factors, trust and perceived risks are critical for explaining mHealth service adoption among Chinese patients.

## Introduction

### Background

Since Professor Robert Istepanian first proposed the concept of *mHealth* in 2005, it has become a new type of medical service mode combining medical services with the internet, medical sensors, mobile equipment, and information communication technology [1]. Compared with traditional medical services, mHealth offers clear advantages, such as portability, mobility, personalization, and ubiquity [2]. The users of mHealth services are diverse, ranging from general public, physicians, and nurses to patients with or without chronic diseases. mHealth services generally consist of Web-based appointments with physicians; Web-based health consultations; health information seeking; and medical examination result checking via wearable, portable devices; and smart devices or smartphone-based apps [3]. Enabled with the mobile devices, health services are available without the constraints of time and space [4,5]. mHealth services cost much less than the traditional ones [6]. In addition, the Chinese government has been implementing the “Health China” strategy since 2016, and the primary objective of this strategy is to solve the problem of “difficulty and expense of seeing a doctor.” Thus, the development and success of mHealth services are important in implementing this national strategy.

mHealth services have garnered extensive attention worldwide [7]. A report released by the World Health Organization in 2011 affirmed that many countries had implemented mHealth care schemes [8]. Moreover, the global telemedicine market was expected to reach 27.3 billion dollars in 2016 [9]. For instance, more than 40,000 health care apps were available in the Apple iTunes Store in 2013 [10,11]. To protect the rights of users, the US Food and Drug Administration has implemented certain laws for mHealth, such as approval, denial, and licensing of medical equipment [11]. mHealth service has flourished in China as well. According to Wang, China’s market of mHealth services will have reached 12.53 billion in 2017 [12]. Enabled by the internet, users can easily communicate their sensitive personal information, such as private diseases or emotions, to others. However, the risk of information leakage can hinder users from utilizing mHealth services [11,13]. Risks incurred by legal concerns may also emerge because no specific law enforcement regulates mHealth services in China.

Although mHealth services can reduce health care costs, improve health care quality, and promote health education, some problems may arouse individual risk perception. Considering the co-existence of technical advantages and potential risks of the mHealth service, we added perceived risk into the classic technology acceptance model (TAM) to analyze the users’ adoption of mHealth services. The TAM represents the positive factors, whereas the perceived risk represents the negative factors associated with mHealth service adoption. In addition, we introduced trust as a middle variable to further explore the beneficial effects of and the risk factors associated with the adoption of mHealth services.

### Literature Review and Hypothesis

#### Literature Review

Studies about mHealth services can be divided into four categories: (1) The current situation of services, which primarily focuses on the status quo of mobile medical services; the development of the services; and the existing defects, problems, or challenges; (2) the technical part of the services, which mainly explores the design and implementation of mHealth service platforms and system terminals; (3) the analysis and evaluation of application effects, which compares the effectiveness of certain mobile medical services and evaluates the effects; and (4) the acceptance of services, which empirically analyzes the factors influencing the consumers’ willingness to adopt mHealth services. For instance, Marzano et al have studied the application of mHealth care in the field of mental health and discussed the associated risks [14]. Liang et al designed an emergency call service, which helped users to quickly and accurately transfer their emergency data to nearby search and rescue personnel through mobile medical social networks [15]. Lv et al used randomized controlled trials to examine the impact of mHealth services that send health messages to patients with asthma on patients’ perceived control [16]. Deng et al also compared the adoption of mHealth services between middle-aged and older users in China [17].

From the perspective of acceptance, Lim et al used the TAM as a theoretical basis to explore the adoption behavior in using mobile phones to search for health information [18]. The results showed that perceived usefulness, perceived ease of use, and self-efficacy significantly affected the adoption. Another research showed that technical anxiety has no significant effect on the willingness to adopt [19]. Wu et al combined TAM and theory of planned behavior to explore the adoption intentions of medical staff toward mobile medical services; the results confirmed that attitude, perceived usefulness, perceived behavioral control, and subjective norms significantly influence the willingness to adopt [20]. Alaiad and Zhou built a model based on the unified theory of acceptance and use of technology (UTAUT) and investigated the willingness to adopt home medical robots for all types of personnel in the organization; the results validated that performance expectations and social effect convenience and conditions significantly affect users’ adoption of home medical robots and that privacy and ethical concerns negatively affect users’ adoption intentions [21].

#### Technology Acceptance Model

Proposed by Davis in 1989, the TAM is a classical theory used in predicting and interpreting users’ adoption of and behavior toward information technology [22]. Similar to the theory of rational behavior and the theory of planned behavior, TAM theory follows the idea of *Faith-Intention-Behavior.* For an individual, one’s faith, for example, attitude and beliefs, affects his or her intention to act in the first place, and then, the actual behavior will change accordingly [23]. The TAM mainly consists of five variables: perceived usefulness, perceived ease of use, attitude, behavioral intention, and actual behavior. According to the TAM, whether an individual performs a certain goal behavior depends on his or her behavioral intention to perform the behavior. Behavioral intention is determined by one’s attitude and perceived usefulness toward a certain behavior or technology, which can also be called adoption intention. In addition, perceived usefulness and perceived ease of use can influence one’s attitude toward a certain behavior or technology [24]. Later studies have proposed new theories based on the TAM, such as the TAM2 and UTAUT [25,26]. Compared with the TAM, TAM2 incorporates the antecedents of perceived usefulness; TAM 3 adds external factors influencing the perceived ease of use; and the UTAUT model combines exploratory factors, performance expectancy, effort expectancy, social influence, facilitating conditions, and four moderators (gender, age, experience, and voluntariness of use). mHealth service involves patients’ health issue, which is irreversible and cannot be returned. If patients use wrong health information, then the body will be greatly affected. Thus, patients’ risks should be considered. In this study, we combined the TAM and patients’ perceived risk concerns to examine their adoption of mHealth services.

#### Perceived Risk

Raymond A. Bauer, a Harvard University scholar in the field of psychology, introduced the concept of perceived risk. Bauer believed that any act of an individual can lead to unforeseen consequences. The undesirable or unexpected aspect of these consequences is that individuals cannot control them and that they may cause some loss to the individual, which is called the risk of an individual’s actions [27]. Many scholars have further defined perceived risk in their research. Cunningham showed that if the final outcome of one’s behavior is unpleasant or perceived detrimental to him or her, the potential loss of this outcome will be perceived risks [28]. Peter and Ryan believed that perceived risk is one’s subjective perception of the expected loss of a target behavior [29]. Mitchell regarded perceived risk as one’s subjective assessment and perception where behavior may cause a loss [30].

When introduced into the field of behavior research, Bauer emphasized the subjectivity of perceived risks rather than focusing on objective risks [27]. Although objective risks persist, only subjective risks perceived by an individual might affect his or her behavior. On the basis of Bauer’s work, several scholars have explored the dimensions of perceived risks. Jacoby and Kaplan classified the perceived risk into 5 types: financial, performance, physical, psychological, and social [31]. Stone and Grønhaug added the time risk into the previous research and proved that the 6 types can interpret the perceived risk at a rate of 88.8% [32]. Of all the classification methods, the 6 types of perceived risk have been generally recognized by the academic community. However, the specific variables of the perceived risk are not limited to the 6 types. Scholars usually group them into different risk variables according to their research context. For instance, Lee studied individuals’ adoption of Web-based banking services from the perspectives of function, time, financial, social, and security risks [33]. Kim et al examined the effect of perceived risks on individuals’ e-commerce purchasing decisions based on 3 dimensions: privacy protection, security protection, and information quality [34]. Although perceived risks are mainly adopted in the context of business, they are being gradually applied to the health care fields, for example, wearable devices and electronic medical records [35,36].

#### Theoretical Foundation and Hypothesis

##### Trust

Trust is an essential factor for attracting new users and maintaining the loyalty of old users [34]. If one trusts the services provided by mHealth, he or she is likely to adopt the service [37]. In this study, we utilized the definition of trust by Doney et al and Yang et al [38,39]. Trust lies on users’ willingness to believe and implement the advices or information acquired through mHealth services; thus, users prefer to believe that mHealth services can fulfill their health needs [38,39]. As a belief variable, trust is an individual’s positive expectation toward another party’s future behaviors [40]. According to the TAM, individuals’ beliefs can influence their attitudes and adoption intentions. If one trusts mHealth and has a positive attitude toward mHealth services, he or she is likely to adopt these services. Therefore, we propose the following hypothesis:

H1: One’s trust toward mHealth services is positively associated with his or her intention to adopt the services.

##### Perceived Usefulness

According to Holden and Karsh, perceived usefulness refers to one’s subjective perception that the use of new technologies or services will improve his or her work efficiency [24]. In this study, perceived usefulness is one’s belief that the use of mHealth services can enhance or improve his or her health condition, which suggests that mHealth services might be useful for individuals to obtain low-cost health information easily and fast, thereby eventually improving the overall health care quality [41]. In the TAM theory, perceived usefulness may exert an influence on one’s attitude and adoption intention [42]. For instance, Lim et al. confirmed that perceived usefulness significantly affected women’s adoption intention of health information seeking using mobile phones [18]. Wu et al proved that medical staff are likely to adopt mHealth services if they perceive it as useful in their daily work [20]. Similar to attitudes, trust is a classic belief variable. According to the TAM theory, perceived usefulness can significantly influence people’s attitudes toward a certain service or technology. If one perceives a service as useful, he or she turns to evaluate it as highly positive [24]. Therefore, we argue that an individual is likely to trust mHealth services if he or she perceives these services as useful. Moreover, an individual is likely to adopt mHealth services if he or she perceives them as useful. Therefore, we propose the following hypotheses:

H2a: Perceived usefulness is positively associated with one’s trust toward mHealth services.

H2b: Perceived usefulness is positively associated with one’s adoption intention toward mHealth services.

##### Perceived Ease of Use

Another commonly used variable in the study on technology adoption behavior is *perceived ease of use*, which has been defined as the perception that using a particular technology will be free from physical or mental efforts [24]. In the mHealth context, perceived ease of use refers to the degree of difficulty experienced during the use of mHealth services. Different from the traditional health services, mHealth is based on the internet and mobile devices that provide health services [43]. Compared with elderly individuals, young individuals are more open to new things and are more likely to perceive mHealth services as easy to use. In the TAM, perceived ease of use has a direct influence on perceived usefulness and attitude [42]. Perceived usefulness is important for one’s adoption intention. An individual will not use mHealth services if he or she perceives them as difficult to use regardless of the provision of usefulness [44]. Therefore, we propose the following hypotheses:

H3a: Perceived ease of use is positively associated with one’s trust toward mHealth services.

H3b: Perceived ease of use is positively associated with one’s adoption intention toward mHealth services.

**Figure 1:**
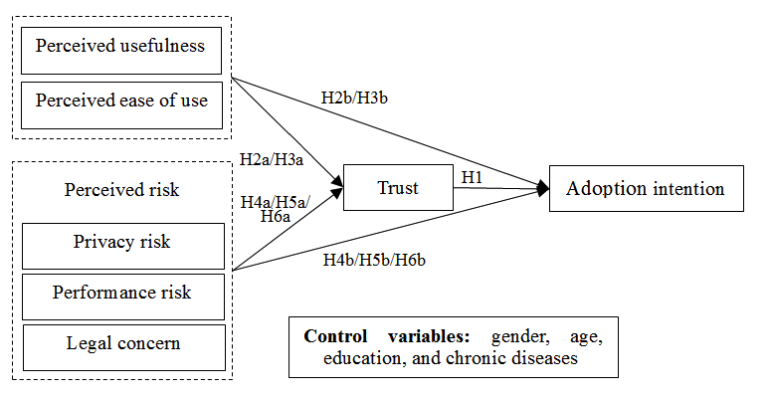
Proposed research model. H: hypothesis.

##### Perceived Risk

*Perceived risk* refers to one’s perception of uncertainty in the use of mHealth services and its severity in terms of consequences [45]. The mHealth service in China is still in its infancy stage, promising but also problematic [24]. Potential problems, such as privacy invasion, may pose a certain risk to users. mHealth services might not work well in terms of desired performance. Meanwhile, legal concern is also highlighted in China’s context, where no specific law enforcement regulates the interest of parties in mHealth services. In this study, we measured the perceived risks through the following 3 variables: privacy risk, performance risk, and legal concern. The measurement of legal concern was shown by Alaiad and Zhou. In their research, they explored the effect of privacy, ethical, and legal concerns on the adoption intention toward home medical robots [21]. In the mHealth context, privacy risk refers to the possibility of information abuse due to the use of mHealth services, such as information theft and leakage [46]. Performance risk refers to the possibility of a match between the desired outcomes and actual use of mHealth services. Legal concern refers to the possibility of users’ worry due to lack of law enforcement [21]. Previous research has also shown that perceived risk and privacy can effectively predict users’ adoption intention and trust [21,34]. We argue that the greater the perceived risk, the lesser the trust and adoption intention toward mHealth services. Therefore, we propose the following hypotheses:

H4a: Privacy risk is negatively associated with one’s trust toward mHealth services.

H4b: Privacy risk is negatively associated with one’s adoption intention toward mHealth services.

H5a: Performance risk is negatively associated with one’s trust toward mHealth services.

H5b: Performance risk is negatively associated with one’s adoption intention toward mHealth services.

H6a: Legal concern is negatively associated with one’s trust toward mHealth services.

H6b: Legal concern is negatively associated with one’s adoption intention toward mHealth services.

##### Control Variables

Adding control variables will increase the explanatory power [47]. The control variables include gender, age, education, and chronic diseases. Patients with chronic diseases, who suffer throughout their lifetime, are more aware of their health condition and need constant health care compared with patients with acute diseases. Thus, we propose that chronic diseases may lead patients to significant mHealth service usage because of the associated convenience. Long-term monitoring of chronic diseases is very important for the treatment and transformation of diseases. We argue that whether users suffer from chronic diseases may affect the adoption intention toward mHealth services to some extent. On the basis of the abovementioned reasons, in this study, we used *chronic diseases* as the control variable.

Considering all the above mentioned hypotheses, we propose a new research model as shown in Figure 1.

## Methods

### Development of the Study Questionnaire

We developed a survey questionnaire for the proposed research model. The measurement items were either directly obtained from extant studies or slightly modified to fit in China’s context. Items for perceived usefulness are from Mun and Hwang and Parkes, and those for perceived ease of use are from Burton-Jones and Hubona [48-50]. Items for privacy risk, performance risk, and legal concern are from Featherman and Pavlou, Nepomuceno et al, and Alaiad and Zhou, respectively [21,51,52]. Items for trust are from Mun et al and Martin et al [53,54] and those for adoption intention are from Wu et al [55].

**Table 1:** Demographic characteristics of the participants (N=388).

Characteristic		Number of participants, n (%)
**Gender**		
	Male	203 (52.3)
	Female	185 (47.7)
**Age (years)**		
	≤20	21 (5.4)
	21-30	116 (29.9)
	31-40	123 (31.7)
	41-50	85 (21.9)
	51-60	32 (8.2)
	≥60	11 (2.8)
**Education level**		
	Junior high school and below	32 (8.2)
	High school	54 (13.9)
	Specialist college	82 (21.1)
	Undergraduate degree	180 (46.4)
	Master’s degree	35 (9.0)
	Doctoral degree and higher	5 (1.3)
**Marital status**		
	Unmarried	64 (16.5)
	Married	288 (74.2)
	Divorced	28 (7.2)
	Widowed	8 (2.1)
**Career**		
	Enterprises (state-owned or foreign or private)	54 (13.9)
	Civil servants	101 (26.0)
	Medical workers	35 (9.0)
	Freelancers	46 (11.9)
	Self-employed	34 (8.8)
	Students	47 (12.1)
	Other	71 (18.3)
**Personal monthly income (Chinese yuan)**		
	≤1000	43 (11.1)
	1001-3000	64 (16.5)
	3001-5000	142 (36.6)
	5001-7000	94 (24.2)
	≥7000	45 (11.6)
**Suffering from chronic diseases**		
	Yes	138 (35.6)
	No	250 (64.4)

To ensure the quality of the designed questionnaire, we performed a pretest among 30 postgraduates and 30 undergraduates in the Tongji Medical College. We assumed that the medical students were young and familiar with mHealth services in general, and they may have had considerable opinions regarding these services. The final questionnaire was developed on the basis of the modifications of the pretest. The questionnaire consists of 34 questions with two sections: (1) 7 questions about demographic information (eg, age, gender, educational level, and chronic diseases) and (2) 27 questions to measure perceived usefulness, perceived ease of use, privacy risk, performance risk, legal concern, trust, and adoption intention. All the items in section 2 were measured using a 5-point Likert scale, with scores ranging from 1 (strongly disagree) to 5 (strongly agree). The details of the questionnaire can be found in Multimedia Appendix 1.

### Data Collection

To test the proposed research model, we conducted an on-site survey in 3 large hospitals in China from September 20 to October 20, 2016. Among them, one is a medical college hospital, where patients comprise the students or faculty members studying or working in the university campus and the other two are famous hospitals in central China, where patients come from all over China, particularly the central region. Both are affiliated with this medical college. The target population included patients and their caregivers, who may be highly interested in mHealth services. We first asked them politely whether they would give 5-10 minutes to participate in our survey. If they answered yes, the survey was conducted accordingly. Small gifts were provided as incentives to those who completed the questionnaire. To avoid disturbing patients, we asked patients and their caregivers either waiting for a doctor or chatting with each other in the hospital waiting and resting areas to fill in our questionnaires; a total of 450 questionnaires were distributed and collected. After discarding the questionnaires with incomplete answers and those with the same answers, we obtained a total of 388 usable responses; the effective response rate was 86.2%. Approximately, 52.3% (203/388) of the respondents were male, and the age of the respondents ranged from 20 to 60 years. Approximately 67.0% (260/388) of the respondents were younger than 40 years and 2.8% (11/388) were older than 60 years. Approximately half of the respondents had a bachelor’s degree or higher, and 35.6% (138/388) of the respondents had at least one chronic disease. Table 1 shows the detailed information of the respondents.

## Results

### Structural Equation Model

The structural equation model includes the measurement and structural models. We analyzed the research model using a two-step approach with an exploratory factor analysis and a confirmatory factor analysis to test the measurement and structural models, respectively [56].

### Results of the Measurement Model Testing

We performed a confirmatory factor analysis to test the measurement equation, including reliability and validity tests. Reliability refers to the degree of reliability of each construct. Composite reliability and Cronbach alpha were used to measure the reliability. Validity is a measure of the validity of a measurement scale, usually based on discriminant validity and convergent validity. Discriminant validity refers to the degree of difference between the items with different latent variables, which can be tested using the average variance extracted of each latent variable and the standard load of each item. Table 2 shows the confirmatory factor analysis results of the measurement model. The composite reliability, Cronbach alpha, and average variance extracted of each construct and all the standard loadings are greater than the recommended values, indicating a good reliability and discriminant validity [56].

Convergent validity refers to a higher level of correlation between the measurement items of the same variable, which can be tested using the square root of the average variance extracted of each construct and its correlation coefficients with other constructs. The correlation coefficients between any two variables are smaller than the square root of the corresponding average variance extracted, thereby indicating a high discriminant validity [57]. Table 3 shows the specific results.

The fitting degree of a model is an evaluation of the research model. The commonly used goodness-of-fit indices are the ratio of χ^2^ and degrees of freedom (df), Root Mean Square Error of Approximation (RMSEA), goodness of fit index (GFI), adjusted goodness of fit index (AGFI), comparative fit index (CFI), mormed fit index (NFI), and incremental fit index (IFI). The confirmatory factor analysis results are presented in Table 2 and Table 4 (χ^2^_388_=840.0, χ^2^/df=2.165<3, RMSEA=0.055<0.08, and AGFI=0.863>0.80). The values of GFI, CFI, NFI, and IFI are all greater than 0.90. All the fitting indices of the research model are above the normal average acceptance level, which shows that the research model agrees well with the acquisition data [58].

### Results of the Structural Model Testing

We used a regression method to test structural equations. The proposed research model includes seven latent constructs and several control constructs. The seven latent constructs are the primary factors, including perceived usefulness, perceived ease of use, privacy risk, performance risk, legal concern, trust, and adoption intention, and they were analyzed in Model 1. The control constructs such as gender, age, education, and chronic diseases were added into Model 2 to test the control effects.

Table 4 summarizes the results of multiple linear regression analysis. Table 5, Table 6, and Table 7 show the specific results of the hypothesis testing. The results showed that trust, perceived usefulness, and perceived ease of use positively correlated with the adoption intention in the context of mHealth services. Meanwhile, privacy and performance risks negatively correlated with trust and adoption intention. We did not observe a significant correlation between legal concern and trust or adoption intention. For the control constructs, education and chronic diseases can be effective predictors for individual adoption intention toward mHealth services. The variations in age lead to different levels of trust toward mHealth services. However, gender has no significant effect either on trust or adoption behavior.

**Table 2:** Confirmatory factor analysis results of the measurement model.

Constructs and items		Standard loadings	Average variance extracted of each latent variable	Composite reliability	Cronbach alpha
**Perceived usefulness**			0.625	0.833	.747
	1	0.76
	2	0.78
	3	0.83
**Perceived ease of use**			0.585	0.875	.765
	1	0.72
	2	0.76
	3	0.74
	4	0.77
	6	0.83
**Privacy risk**			0.720	0.911	.909
	1	0.81
	2	0.86
	3	0.91
	4	0.81
**Performance risk**			0.584	0.848	.846
	1	0.68
	2	0.79
	3	0.84
	4	0.74
**Legal concern**			0.672	0.857	.843
	1	0.63
	2	0.89
	3	0.91
**Trust**			0.612	0.887	.877
	1	0.75
	2	0.80
	3	0.81
	4	0.80
	5	0.75
**Adoption intention**			0.657	0.851	.853
	1	0.85
	2	0.80
	3	0.78

**Table 3:** Correlation coefficient matrix and square root of average variance extracted of latent variables.

Constructs	Perceived usefulness	Perceived ease of use	Privacy risk	Performance risk	Legal concern	Trust	Adoption intention
Perceived usefulness	0.790^a^	—^b^	—	—	—	—	—
Perceived ease of use	0.401	0.765^a^	—	—	—	—	—
Privacy risk	−0.121	−0.058	0.848^a^	—	—	—	—
Performance risk	−0.086	−0.212	0.155	0.764^a^	—	—	—
Legal concern	−0.069	−0.144	0.316	0.412	0.819^a^	—	—
Trust	0.310	0.321	−0.288	−0.227	−0.245	0.782^a^	—
Adoption intention	0.440	0.233	−0.247	−0.185	−0.119	0.438	0.810^a^

^a^Square root of average variance extracted.

^b^Not applicable.

**Table 4:** Fit index of the research model.

Fit	χ^2^/df^a^	RMSEA^b^	GFI^c^	AGFI^d^	CFI^e^	NFI^f^	IFI^g^
Recommended value	<3	<0.08	>0.90	>0.80	>0.90	>0.90	>0.90
Research model	2.165	0.055	0.909	0.863	0.925	0.905	0.935

^a^χ^2^/df: chi-square divided by degrees of freedom.

^b^RMSEA: Root Mean Square Error of Approximation.

^c^GFI: goodness of fit index.

^d^AGFI: adjusted goodness of fit index.

^e^CFI: comparative fit index.

^f^NFI: normed fit index.

^g^IFI: incremental fit index.

**Table 5:** Results of multiple linear regression analysis for independent variable Trust. R^2^: coefficient of determination, ΔR^2^: change in the coefficient of determination, ΔF: change in the F-statistic.

Independent variable		Trust			
		Model 1 (95% CI)	*P* value	Model 2 (95% CI)	*P* value
**Independent variable**					
	Perceived usefulness	0.046 (−0.085 to 0.178)	.65	0.042 (−0.090 to 0.174)	.42
	Perceived ease of use	0.027 (−0.116 to 0.170)	.45	0.026 (−0.117 to 0.169)	.60
	Privacy risk	−0.153 (−0.269 to −0.037)	.005	−0.148 (−0.267 to −0.032)	.03
	Performance risk	−0.147 (−0.264 to −0.030)	.002	−0.121 (−0.239 to −0.004)	.01
	Legal concern	−0.033 (−0.061 to 0.156)	.52	−0.033 (−0.096 to 0.121)	.40
**Control variables**					
	Gender	—^a^	—	0.044 (−0.073 to 0.191)	.38
	Age	—	—	0.150 (0.084 to 0.216)	.008
	Education	—	—	0.092 (−0.002 to 0.152)	.12
	Chronic diseases	—	—	0.082 (−0.037 to 0.236)	.81
R^2^		0.280	—	0.313	—
ΔR^2^		—	—	0.033	—
ΔF		—	—	32.447	0.03

^a^Not applicable.

**Table 6:** Results of multiple linear regression analysis for dependent variable Adoption Intention. R^2^: coefficient of determination, ΔR^2^: change in the coefficient of determination, ΔF: change in the F-statistic.

Dependent variable		Adoption intention			
		Model 1 (95% CI)	*P* value	Model 2 (95% CI)	*P* value
**Independent variables**					
	Trust	0.427 (0.327 to 0.527)	<.001	0.371 (0.270 to 0.472)	<.001
	Perceived usefulness	0.125 (0.05 to 0.20)	.02	0.120 (0.008 to 0.232)	.02
	Perceived ease of use	0.107 (0.035 to 0.179)	.01	0.107 (0.012 to 0.202)	.02
	Privacy risk	−0.134 (−0.24 to −0.028)	.004	−0.132 (−0.238 to −0.026)	.002
	Performance risk	−0.252 (−0.369 to −0.135)	.003	−0.221 (−0.339 to −0.103)	.001
	Legal concern	−0.033 (−0.061 to 0.156)	.52	−0.033 (−0.096 to 0.121)	.40
**Control variables**					
	Gender	—^a^	—	0.066 (−0.066 to 0.197)	.08
	Age	—	—	0.027 (−0.039 to 0.093)	.36
	Education	—	—	0.104 (0.044 to 0.164)	.04
	Chronic diseases	—	—	0.112 (0.04 to 0.220)	.05
R^2^		0.460	—	0.512	—
ΔR^2^		—	—	0.052	—
ΔF		—	—	27.593	<.001

^a^Not applicable.

**Table 7:** Results of the hypothesis testing.

Hypothesis	Path	Path coefficient	*P* value	Supported
H1	Trust → Adoption intention	0.427	<.001	Yes
H2a	Perceived usefulness → Trust	0.046	.27	No
H2b	Perceived usefulness → Adoption intention	0.125	.04	Yes
H3a	Perceived ease of use → Trust	0.027	.07	No
H3b	Perceived ease of use → Adoption intention	0.107	.11	Yes
H4a	Privacy risk → Trust	−0.153	.004	Yes
H4b	Privacy risk → Adoption intention	−0.134	.002	Yes
H5a	Performance risk → Trust	−0.147	.003	Yes
H5b	Performance risk → Adoption intention	−0.252	.002	Yes
H6a	Legal concerns → Trust	−0.033	.54	No
H6b	Legal concerns → Adoption intention	−0.065	.08	No

## Discussion

### Principal Findings

With the introduction of perceived risks in the classic TAM theory, this study aimed to explore the factors influencing an individual’s trust and adoption intention toward mHealth services. Furthermore, we analyzed the effects of gender, age, and chronic diseases as control variables. We proposed 11 hypotheses, of which 7 are supported. The primary findings are summarized as follows.

First, trust, perceived usefulness, and perceived ease of use are strong predictors for the adoption intention toward mHealth services, whereas the influence of perceived usefulness and perceived ease of use on trust is not significant. Trust, perceived usefulness, and perceived ease of use are important positive and technical factors explaining a user’s adoption intention toward mHealth services. Among them, trust exerts the greatest influence, followed by perceived usefulness and perceived ease of use. In sum, this result is consistent with those of previous studies by Gu et al and Huang et al [44,59]. It reveals that if individuals think that mHealth services are trustworthy, they are more willing to adopt them. For mHealth service operators, building trust is crucial. Corroborating the results of a study by Zhang et al [60], we also validated that although perceived usefulness and perceived ease of use are still influential in explaining the adoption of mHealth services, their significance seems to decline. In our study, trust significantly correlated with mHealth adoption intention with a *P* value of <.001, whereas perceived usefulness and perceived ease of use significantly correlated with mHealth adoption intention with a *P* value between .05 and .01. This may have been caused by the evolution of the technology itself [61]. People are generally more technology savvier than their counterparts 10 years ago, and they can easily adjust with using modern mHealth services. However, for the mHealth service operators, strategies such as improving service quality and simplifying user process can still result in more individuals adopting the services.

Second, the 3 dimensions of perceived risk, privacy risk, and performance risk negatively correlated with the trust and adoption intention, whereas legal concern showed no significant effect. When people perceive a potential risk associated with using mHealth services, for example, privacy or information leakage issues, they are less likely to trust and adopt these services. This result is similar to that of other studies. For example, Guo et al concluded that privacy concerns significantly influence a user’s trust toward mHealth [13]. Zhang et al also found that privacy concern can affect adoption intention via attitude [62]. Meanwhile, the influence of privacy risk and performance risk on trust and adoption intention differs. Compared with performance risk (−0.147), privacy risk (−0.153) has a more remarkable effect on trust. Compared with privacy risk (−0.134), performance risk (−0.252) has a more remarkable effect on adoption intention. This may be caused by the differentiations of specific risk. For example, performance risk features the perception of the possible risks of specific service functions and quality, and adoption intention centers on people’s willingness to use a certain service. According to previous studies, the function and quality of mHealth services are more likely to affect people’s willingness to adopt these services [18,20]. Privacy risk concerns personal health-related information during the use of mHealth services. Privacy risk is not directly correlated with the functions of the mHealth services and is aroused by psychological factors. Hence, compared with performance risk, privacy risk may likely exert more effect on trust. For mHealth service operators, the major tasks include the following: (1) improving overall quality and (2) building a reliable information security system that protects a user’s privacy [63,64]. These strategies will enhance the trust level of users and will ultimately encourage the adoption of the services. In addition, legal concern is seldom discussed because the primary function of mHealth services in China is Web-based health consultation, and patients still need to obtain medical treatment in hospitals. This may also help us understand the result showing no significant effect of legal concern on trust and adoption.

Third, control variables, such as age and chronic diseases, correlated with trust and adoption intention toward mHealth services. In terms of age, older individuals are more likely to trust mHealth services compared with the young individuals. This result is in accordance with that of several studies. For example, Guo et al found that the influence of personalization and privacy concerns on trust toward mHealth was different between old and young individuals [13]. Morris and Venkatesh also concluded that young individual’s attitudes had a greater influence on their decisions regarding the use of technology, whereas the individuals were more easily influenced by subjective norms and perceived adoption control [65]. In terms of chronic diseases, patients with chronic disease were more likely to adopt mHealth services compared with those without them. A possible explanation is that chronic condition usually requires patients to monitor their health instantly, and mHealth services perform well in addressing this problem. This finding is also consistent with that of an earlier study, which showed that a respondent’s health status can moderate the relationship between trust and an individual’s intention of using mHealth [37].

### Implications and Limitations

On the basis of the TAM and perceived risks, we built an mHealth service adoption model and further tested the model via a cross-sectional study on patients from 3 large hospitals in China. This study has several implications. First, we empirically tested the effects of perceived usefulness, perceived ease of use, privacy risk, performance risk, and legal concern on trust and adoption intention toward mHealth services. The findings supported the effect of perceived risk, trust, and adoption intention on the use of mHealth services. Second, we added several control variables to the research model, which include gender, age, and chronic diseases, and results showed that age and chronic diseases affect an individual’s trust and adoption intention. Although the influence of age and gender on people’s technology adoption behavior has been extensively studied, only few studies have focused on the effects of chronic diseases. This study may serve as a valuable reference for future studies on the effect of chronic diseases on the adoption of mHealth services in China. Third, the empirical results highlight the significant effect of perceived usefulness and perceived ease of use on an individual’s adoption intention and the significant influence of privacy risk and performance risk on trust and adoption intention. These findings may offer practical suggestions for the developers of mHealth services as well as the enterprises working in the mHealth industry. For example, the flow of the mHealth services can be simplified or specific tutorials or videos can be provided. Regarding the risk concerns, the security issue is always the focus of reducing the potential risk perceived by the users.

The study has its own limitations. First, this research was conducted in the context of China’s mHealth services. Thus, the results may not be generalized to other countries and regions. Second, the proposed research model was based on the TAM and perceived risk, and the variance rate explained in the model was 48.9%. Other important factors that are associated with the adoption intention toward mHealth may have been overlooked in this study. Future research can incorporate relevant variables to increase the explanatory power of the research model. Another limitation of this study is its small sample size. We might not have a representative sample of patients who intended to use mHealth services in China. Researchers should exercise caution when citing our results.

### Conclusions

This study proposed an extended TAM research model using the concept of perceived risk to study the determinants of trust and adoption intention toward mHealth. The results corroborated that trust, perceived usefulness, and perceived ease of use positively correlated with adoption intention. Privacy and performance risks negatively correlated with trust and adoption intention toward mHealth services. In terms of the control variables, we confirmed that age has a significant influence on an individual’s trust and that chronic diseases can be an important predictor for mHealth service adoption. These findings are conducive to future research on mHealth service adoption.

## Appendix

Multimedia Appendix 1 Questionnaire: Measurement of the major constructs.

## Abbreviations

AGFI: adjusted goodness of fit index
CFI: comparative fit index
GFI: goodness of fit index
IFI: incremental fit index
NFI: normed incremental fit index
RMSEA: Root Mean Square Error of Approximation
TAM: technology acceptance model
UTAUT: unified theory of acceptance and use of technology
