# Supplementary material for: What Predicts Patients’ Adoption Intention Toward mHealth Services in China: Empirical Study
Source: JMIR Mhealth Uhealth. 2018 Aug 29;6(8):e172. doi: 10.2196/mhealth.9316 (PMC6135967; doi:10.2196/mhealth.9316)
Supplement: Multimedia Appendix 1 [file mhealth_v6i8e172_app1.pdf]

Multimedia Appendix

Questionnaire: Measurement of the major constructs

Perceived usefulness

PU1: I believe using mHealth services can improve the efficiency of health care in general.

PU2: Compared with the traditional offline office visits, I think the communication between physicians and patients over the mobile devices is much better.

PU3: Overall, I think mHealth services are useful for health management.

Perceived ease of use

PEOU1: I do not think using mHealth services for health consultation is difficult.

PEOU2: It is easy for me to learn how to use mHealth services.

PEOU3: The user interface of mHealth services is clear, understandable, and easy to operate.

PEOU4: For sensitive health problems, I think online health consultation is much easier than that of face-to-face consultation.

PEOU5: In general, I think mHealth services are easy to use.

Privacy risk

PVR1: I am afraid that mHealth providers cannot guarantee the confidentiality of user information.

PVR2: I am worried that my personal privacy information will be used for other purposes if I use mHealth services.

PVR3: I am worried that when using mHealth services, my personal information will be abused by cyber criminals.

PVR4: Because of security issue, I am worried about personal information leakage when I consult a doctor about sensitive health problems online.

Performance risk

PER1: I am worried that using mHealth services cannot satisfy my health needs.

PER2: I am afraid that the health advice from mHealth services cannot well address my health concerns.

PER3: Compared with the traditional offline office visits, I am concerned about the quality of online health consultation.

PER4: I am worried that the services provided by mHealth may not match my expectations.

Legal concern

LEC1: I am worried that my personal health-related information is not protected by law when using mHealth services.

LEC2: I am worried that the behaviors of doctors and patients on the internet lack special legal restrictions and may have adverse effects.

LEC3: I am afraid that the rights and interests of users cannot be ensured because of the lack of specific law enforcement on the mHealth services.

Trust

TRU1: Generally, I think doctors on mHealth service platforms are trustworthy.

TRU2: Most of the doctors on the mHealth services platforms are health experts in their field, and I have no doubt about their profession.

TRU3: Doctors in professional health care websites are verified, and I think their credibility is guaranteed.
TRU4: Health consultations with doctors online is reliable to solve my health problems.
TRU5: In general, I trust health advices or tips from doctors on the internet.

Adoption intention

AI1: When I face health problems, I guess I will use mHealth services to solve them.
AI2: I am willing to use mHealth services for health consultation, such as disease control.
AI3: I intend to use mHealth services regularly.
